# Preparation and Performance Study of Carboxylated Nitrile Rubber Based on Phase Transfer Catalysis: Screening of Optimal Catalyst System

**DOI:** 10.3390/polym18070830

**Published:** 2026-03-28

**Authors:** Hongbing Zheng, Dongmei Yue

**Affiliations:** Key Laboratory of Beijing City on Preparation and Processing of Novel Polymer Materials, Beijing University of Chemical Technology, Beijing 100029, China; zhenghongbing@petrochina.com.cn

**Keywords:** XNBR, phase transfer catalysis, catalyst screening, TBAB, carboxyl content

## Abstract

This study systematically screened twelve phase transfer catalysts from three categories, namely ammonium-based compounds, phosphonium-based compounds, and polyethylene glycols of different molecular weights, for the preparation of carboxylated nitrile rubber (XNBR) via phase transfer catalytic hydrolysis. The carboxyl content of the resulting XNBR was quantitatively determined by titration, revealing significant variations with catalyst structure ranging from 0 to 2.2 wt%. Phosphonium catalysts exhibited the highest carboxylation efficiency, with TBPB achieving 2.2 wt%, while ammonium catalysts showed structure-dependent performance, with TBAB reaching 1.1 wt%. PEG catalysts demonstrated optimal efficiency at intermediate molecular weights, with PEG-300 achieving 0.8 wt% and PEG-600 achieving 0.6 wt% but suffered from residual contamination. Through comprehensive evaluation of catalytic efficiency, reaction controllability, safety, and product purity, tetrabutylammonium bromide (TBAB) was identified as the optimal catalyst, achieving the best balance between carboxyl content (1.1 wt%), mild reaction kinetics, minimal catalyst residue, and product uniformity. Using TBAB as the catalyst, XNBR with low (1.1%) and high (3.1%) carboxyl contents were successfully prepared by controlling reaction time. The research demonstrated that carboxyl content had a decisive impact on vulcanization characteristics, mechanical properties, and thermal stability of XNBR. As carboxyl content increased, crosslink density significantly increased, leading to marked improvement in tensile stress at given elongation, tensile strength, and hardness, while elongation at break showed a decreasing trend. Thermogravimetric analysis demonstrated that carboxyl group introduction effectively enhanced the thermal stability of the material. This study provides an important theoretical basis and practical guidance for regulating the carboxylation degree through catalyst molecular design and preparing XNBR with excellent comprehensive performance.

## 1. Introduction

Nitrile butadiene rubber (NBR) is a synthetic rubber produced by emulsion copolymerization of butadiene (CH_2_=CH—CH=CH_2_) and acrylonitrile (CH_2_=CH—CN) as basic structural units. The polar cyano group (-C≡N) in its molecular chain endows it with excellent oil resistance, wear resistance, air tightness, and relatively high mechanical strength [1]. These properties make NBR an irreplaceable key material in industries such as automotive, aerospace, petrochemicals, and sealing products [2]. For instance, in the automotive industry, NBR is widely used in fuel hoses, hydraulic seals, O-rings, and transmission diaphragms; in industrial applications, it is often used to manufacture oil-resistant rollers, conveyor belts, and various dynamic sealing products. Additionally, NBR possesses certain resistance to chemical media and good processing performance, further expanding its application range [3].

However, the non-polar nature of the traditional NBR molecular chain leads to poor compatibility with polar materials [4], and it has inherent limitations in bonding performance and anti-static properties [5,6], which, to some extent, restricts its application expansion in emerging fields such as high-performance composite materials. To overcome these deficiencies, chemical modification of NBR to introduce polar functional groups into its molecular chain has become a key research direction. Among them, the partial conversion of the nitrile group (-CN) to the carboxyl group (-COOH) through hydrolysis to prepare carboxylated nitrile rubber (XNBR) [7] is one of the most concerned modification paths.

XNBR is an important modified variety of NBR, which can be obtained by copolymerizing butadiene, acrylonitrile, and a third monomer containing carboxyl groups (such as acrylic acid or methacrylic acid) [8,9,10]. This structural modification significantly enhances the polarity of the material, endowing it with superior mechanical properties, oil resistance, solvent resistance, and multiple vulcanization cross-linking methods, broadening its application range. Compared with NBR, the introduction of functional carboxyl groups makes XNBR possess better oil resistance, heat resistance, and bonding performance. Moreover, the introduction of active carboxyl groups enables XNBR to be cross-linked not only through the vinyl groups in the butadiene structure but also through ionic or coordination bonds [11,12,13]. The insertion of acidic groups into the rubber provides additional cross-linking, higher continuous use temperature, better tensile properties, good hardness, and chemical resistance. This specific type of NBR exhibits better tear and wear resistance, making it suitable for dynamic applications such as wiper blades and rod seals [14]. XNBR with ionic cross-linking exhibits thermoplastic elastomeric behavior, allowing the rubber to be melt-processed at high temperatures when the ionic cross-linking points dissociate, and the cross-linking points recover upon cooling [4,15].

The preparation technology of XNBR mainly falls into two categories: one is the direct synthesis method, i.e., the terpolymerization of butadiene, acrylonitrile, and carboxyl-containing monomers (such as acrylic acid or methacrylic acid) [16]; the other is the post-modification method, among which the selective hydrolysis of the nitrile groups in ordinary NBR to introduce carboxyl groups is a promising technical path [17]. In the hydrolysis reaction of NBR, a core challenge is the mass transfer efficiency between reactants. The hydrophobic rubber phase and the hydrolysis reagent (such as OH^−^) in the aqueous phase have difficulty in effective contact, which is an important reason for the low efficiency and poor selectivity of traditional hydrolysis methods [18,19,20].

Phase transfer catalysis (PTC) as an effective means to achieve reaction between water-soluble nucleophiles and hydrophobic rubbers was first proposed by Nicholas et al. in 1982 for the desulfurization of rubber [21]. The mechanism of this process involves using quaternary ammonium salts (Q^+^Cl^−^) as phase transfer catalysts to facilitate the migration of anions (X^−^) originally present in the aqueous phase to the organic phase for reaction. The introduction of carboxyl groups can significantly enhance the strength, adhesion, interaction with fillers, and anti-wet skid performance of rubber. However, traditional hydrolysis methods often face severe challenges: direct hydrolysis under strong acid or strong base conditions results in a heterogeneous reaction system with low efficiency and is prone to side reactions such as degradation and cross-linking of the main chain double bonds [22], leading to rubber molecular chain breakage or excessive gel content, making it difficult to control the structure and performance of the final product.

Phase transfer catalysis (PTC) technology offers a novel solution to this problem. This technology enables reactants in two immiscible phases (such as the oily rubber phase and the aqueous phase) to come into contact and react across the phase interface with the help of a catalyst. Applying PTC to the hydrolysis of NBR is expected to achieve efficient and selective conversion of nitrile groups under mild reaction conditions, effectively inhibiting attacks on the unsaturated main chain, and thus precisely controlling the carboxyl content, distribution, and molecular structure of the product.

The efficiency of phase transfer catalysts is significantly influenced by their molecular structure, including alkyl chain length, central atom type, and anion type. Long alkyl chain catalysts (such as CTAB, THABr) exhibit high initial catalytic efficiency due to enhanced lipophilicity, but excessively long chains may lead to catalyst retention in the organic phase, hindering continuous OH^−^ transport and causing steric hindrance effects. Phosphonium catalysts (such as TBPB) show superior catalytic activity compared to analogous ammonium salts due to the higher polarizability of phosphorus atoms; however, their reaction process is more violent, prone to localized overheating and runaway reactions, posing certain safety risks. PEG catalysts with optimal molecular weight (PEG-600/1500) demonstrate significant catalytic effects, yet catalyst removal from the product proves difficult, with residual PEG potentially affecting material purity and long-term stability. Therefore, systematic screening of catalyst systems and identification of the optimal catalyst are crucial for developing an efficient, controllable, and safe preparation method for XNBR.

**Figure 1:**
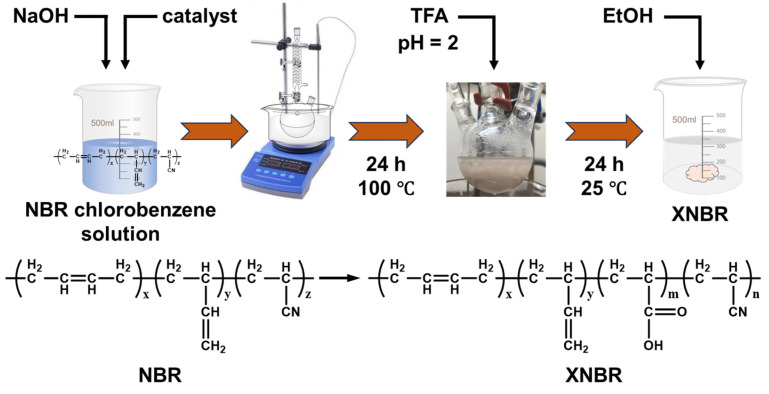
Schematic representation of the carboxylation of nitrile rubber (NBR) to carboxylated nitrile rubber (XNBR) via phase transfer catalytic hydrolysis.

Based on this, this study proposes to prepare carboxylated NBR using the phase transfer catalytic hydrolysis method, with systematic screening of twelve catalysts across three categories (Figure 1). The aim of this work is to comprehensively investigate the influence of catalyst molecular structure on hydrolysis efficiency, product microstructure, and final performance, to identify the optimal catalyst system, and to develop a new green synthesis process for XNBR that is mild in conditions, highly controllable, and yields excellent product performance, providing a new theoretical basis and technical approach for the preparation of high-performance specialty rubber.

## 2. Materials and Methods

### 2.1. Materials

NBR (with 33% acrylonitrile content) was purchased from Lanzhou Petrochemical Company (Lanzhou, China). Chlorobenzene (98%), trifluoroacetic acid (TFA, 30%), sodium hydroxide (99%), tetrabutylammonium bromide (TBAB, AR), cetyltrimethylammonium bromide (CTAB, AR), tetrahexylammonium bromide (THABr, AR), tetraethylammonium bromide (TEAB, AR), tetrabutylammonium chloride (TBAC, AR), phosphonium-based catalysts (tetrabutylphosphonium bromide (TBPB, AR), tributyl(octyl)phosphonium bromide (TBOPB, AR), tributylhexadecylphosphonium bromide (TBHDPB, AR), and polyethylene glycols (PEG-300, PEG-600, PEG-1500, PEG-3000, AR) were all purchased from Shanghai Titan Scientific & Technology Co., Ltd. (Shanghai, China).

### 2.2. Synthesis of Carboxylated NBR (XNBR)

A mixture of NaOH aqueous solution (1.0 M, 40 mL) and chlorobenzene (25 mL) was charged with NBR (1.0 g) and the corresponding phase transfer catalyst (0.1 mmol). The flask was placed in a preheated oil bath at 100 °C and stirred. For each catalyst, the reaction was carried out for 24 h to prepare XNBR. After cooling to room temperature, the aqueous phase was acidified with trifluoroacetic acid to pH = 2 and stirred at room temperature for an additional 24 h. The product was then washed with ethanol and dried to obtain a light yellow solid compound.

Using TBAB as the catalyst and controlling the reaction time (24 h and 48 h), two types of XNBR with different carboxyl contents were prepared: low carboxyl content XNBR (IX-NBR, obtained after 24 h) and high carboxyl content XNBR (HX-NBR, obtained after 48 h).

## 3. Results and Discussion

### 3.1. Structural Characterization of XNBR Prepared by Twelve Catalysts

To quantitatively evaluate the catalytic efficiency of different phase transfer catalysts, the carboxyl content of XNBR prepared with each catalyst was determined by titration analysis, and the results are summarized in Table 1. The carboxyl content varied significantly with catalyst type and structure, ranging from 0 to 2.2 wt% [23], indicating that catalyst molecular architecture plays a decisive role in the hydrolysis efficiency of nitrile groups.

Phosphonium catalysts demonstrated the highest carboxylation efficiency among all three categories, with TBPB achieving 2.2 wt% carboxyl content, followed by TBOPB (1.4 wt%) and TBHDPB (1.2 wt%). This superior performance can be attributed to the stronger polarizability of phosphorus atoms compared to nitrogen, which enhances their ability to form loose ion pairs with OH^−^ and transfer them effectively to the organic phase. However, despite their high catalytic efficiency, phosphonium catalysts exhibited violent and exothermic reaction behavior during synthesis, with a tendency for localized overheating and runaway reactions. This poses significant safety concerns for practical scale-up applications and requires stringent reaction control measures.

Ammonium catalysts exhibited a clear structure-dependent trend in carboxyl content. Short-chain TEAB, with its insufficient lipophilicity due to the very short C2 alkyl chain, yielded only 0.2 wt% carboxyl content, indicating poor phase transfer efficiency as the catalyst cannot effectively enter the organic phase. Long-chain ammonium catalysts showed unexpectedly low carboxyl contents: THABr (C6) produced 0 wt% carboxyl content, while CTAB (C16) achieved only 0.7 wt%. This paradoxical result can be explained by catalyst over-retention in the organic phase: excessively long alkyl chains enhance initial lipophilicity but hinder the return of the catalyst to the aqueous phase after ion exchange, disrupting the catalytic cycle. Furthermore, the long chains may create steric hindrance that impedes OH^−^ transport to remaining nitrile sites and physically block access to reactive centers. TBAB, with its moderate C4 chain length, achieved a balanced performance with 1.1 wt% carboxyl content, demonstrating effective phase transfer capability without excessive retention. The influence of the counter anion was also evident when comparing TBAB and TBAC: the bromide salt (TBAB) yielded higher carboxyl content (1.1 wt%) than the chloride salt (TBAC, 0.5 wt%), consistent with the trend of leaving group ability (I^−^ > Br^−^ > Cl^−^) in phase transfer catalytic reactions.

PEG catalysts showed optimal performance at intermediate molecular weights. PEG-300 achieved 0.8 wt% carboxyl content, while PEG-600 reached 0.6 wt%. Higher molecular weight PEG-1500 and PEG-3000 showed diminished efficiency with 0.2 and 0 wt% carboxyl content, respectively, likely due to increased viscosity and steric hindrance limiting the accessibility of catalytic sites. Lower molecular weight PEG (PEG-300) may not provide sufficient chain length for effective cation complexation. However, an important limitation of all PEG catalysts is the difficulty of complete removal from the XNBR product, as confirmed by subsequent spectroscopic and thermal analyses, with residual PEG potentially affecting material purity and long-term stability.

### 3.2. Spectroscopic Characterization of XNBR Prepared by Twelve Catalysts

**Figure 2:**
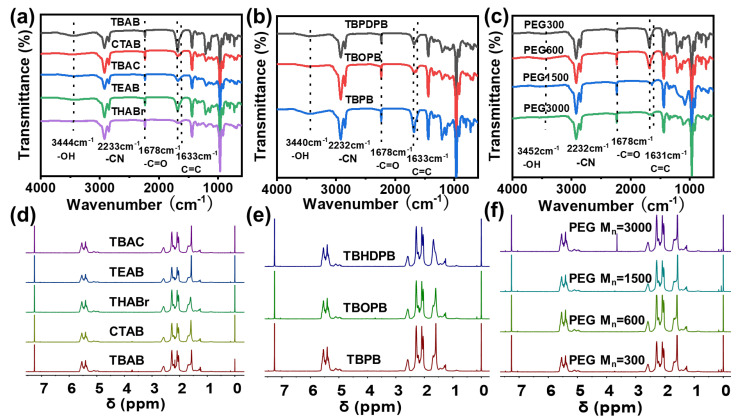
(**a**) FTIR spectra of ammonium-catalyzed XNBR; (**b**) FTIR spectra of phosphonium-catalyzed XNBR; (**c**) FTIR spectra of PEG-catalyzed XNBR; (**d**) ^1^H-NMR spectra of ammonium-catalyzed XNB; (**e**) ^1^H-NMR spectra of phosphonium-catalyzed XNBR; (**f**) ^1^H-NMR spectra of PEG-catalyzed XNBR.

To investigate the effects of different catalyst systems on the structure of XNBR, Fourier Transform Infrared Spectrometer (FTIR) and nuclear magnetic resonance spectrometer (^1^H-NMR) analyses were conducted on the samples obtained from different catalytic hydrolysis processes. The infrared spectra of all products showed characteristic peaks of the nitrile group (-C≡N) from the NBR main chain at 2232 cm^−1^ (Figure 2a–c), confirming the retention of the main chain structure. More importantly, all spectra exhibited two key characteristic absorption peaks representing carboxyl groups (-COOH): broadened absorption bands around 3440 cm^−1^ corresponding to the stretching vibration of hydroxyl groups (-O-H), and clear absorption peaks around 1678 cm^−1^ corresponding to the stretching vibration of the carboxylic acid carbonyl group (C=O) [24]. The simultaneous appearance of these two characteristic peaks is a universal indicator of successful carboxylation, proving that all three catalyst categories can effectively catalyze the hydrolysis reaction and introduce carboxyl groups onto the NBR chain.

Although carboxylation was successful in all cases, the subtle differences in the infrared spectra clearly reflect the efficiency and characteristics of different catalysts. In ammonium catalysts (TEAB, TBAB, THABr, CTAB) (Figure 2a) and phosphonium catalysts (TBPB, TBOPB, TBHDPB) (Figure 2b), XNBR prepared by catalysts with longer alkyl chains (such as THABr, CTAB, TBHDPB) showed stronger carbonyl peaks at 1700 cm^−1^ than their shorter-chain counterparts (such as TEAB) [25]. This is attributed to longer alkyl chains enhancing the lipophilicity of catalysts, allowing better compatibility and dispersion in the NBR matrix, thereby improving contact efficiency with reaction sites. However, careful analysis reveals that excessively long alkyl chains (C16 for CTAB, C6 for THABr) may lead to catalyst over-retention in the organic phase, hindering continuous OH^−^ transport and creating steric hindrance effects. This is reflected in the broader molecular weight distributions observed in GPC analysis for these samples, suggesting less uniform reaction outcomes.

A comparison between phosphonium and ammonium catalysts with similar alkyl chain lengths (e.g., TBPB vs. TBAB) revealed that the phosphonium catalysts exhibited stronger carbonyl peak intensities, suggesting higher catalytic activity. This enhanced performance can be attributed to the larger atomic radius and stronger polarizability of the phosphorus atom compared to nitrogen, which renders phosphonium salts more effective as phase transfer catalysts. However, this higher activity was accompanied by more violent reaction processes with a tendency for localized overheating and runaway reactions, posing safety concerns and necessitating stricter reaction control. The influence of the counter anion was also investigated by comparing the catalytic performance of TBAB and TBAC. The bromide salt (TBAB) yielded products with slightly higher carbonyl peak intensity than the chloride salt (TBAC), which is consistent with the established trend of leaving group ability (I^−^ > Br^−^ > Cl^−^) in phase transfer catalytic reactions.

In the PEG series catalysts (PEG-300, PEG-600, PEG-1500, PEG-3000) (Figure 2c), the absorption intensity of the carbonyl peak at 1678 cm^−1^ shows a trend of first increasing and then decreasing with molecular weight. PEG-600 and PEG-1500 exhibit the most significant carbonyl peak intensities, while PEG-300 and PEG-3000 show relatively weaker intensities. This clearly indicates that there is an optimal range for PEG molecular weight. When the molecular weight is too low (PEG-300), the chain segment structure may not effectively stabilize reaction intermediates; when the molecular weight is too high (PEG-3000), increased steric hindrance and higher viscosity may limit the accessibility of catalytically active sites. However, an important limitation of PEG catalysts is the difficulty of complete removal from the XNBR product. Residual PEG can affect material purity and long-term thermal stability, as evidenced by TGA analysis showing additional low-temperature weight loss steps for PEG-catalyzed samples.

Comprehensive FTIR analysis reveals that TBAB exhibits well-balanced performance: its carbonyl peak intensity is moderately strong, comparable to or slightly higher than other catalysts with similar chain lengths, while its reaction process is mild and controllable. This positions TBAB as the catalyst achieving the optimal balance between catalytic efficiency and reaction controllability.

In the ^1^H-NMR spectrum of unmodified NBR (Figure S1b), multiple signals can be observed between 2.0 and 3.0 ppm [26]. The signal around 2.0–2.3 ppm corresponds to the methylene protons (–CH_2_–) adjacent to the cyano group (–C≡N) in the acrylonitrile units, while the signal around 2.3–2.5 ppm is attributed to the methine proton (–CH–CN) directly attached to the cyano group. Additionally, methylene protons in the α-position relative to the double bond (from 1,4-polybutadiene units) also contribute to the signals in the 2.0–2.5 ppm region. The broad signals in the 1.0–1.5 ppm region correspond to methylene (–CH_2_–) protons on the polymer main chain. It should be noted that no methyl (–CH_3_) groups are present in the unmodified NBR polymer chain, consistent with its copolymer structure derived from butadiene and acrylonitrile [27].

For ammonium-catalyzed XNBR samples (Figure 2d), a strong signal appears at 3.0–3.5 ppm, which is typical of the chemical shift of methyl or methylene protons (-N^+^-CH_3_/-CH_2_-) directly connected to the quaternary nitrogen cation (N^+^), confirming the incorporation or residual presence of quaternary ammonium salt structures. The samples obtained from the CTAB catalyst show extremely strong signals at 0.9 ppm (terminal methyl, -CH_3_) and 1.2–1.3 ppm (long-chain methylene, (CH_2_)_n_) (Figure 2d), which is clear evidence of the long alkyl chain (C16). The signal intensities of THABr (C6) and TBAB (C4) at the same positions decrease successively, consistent with the shortening of alkyl chain length (C6 → C4). TEAB (C2) shows very weak signals in the 1.2–1.3 ppm region, as its alkyl chain is the shortest. These systematic changes effectively verify the successful incorporation of each quaternary ammonium salt’s molecular structure into or onto NBR. Notably, the stronger residual signals for long-chain catalysts (CTAB, THABr) indicate higher catalyst retention, which may affect product purity.

In the ^1^H-NMR spectra of phosphonium-catalyzed XNBR (Figure 2e), signals of methylene protons directly connected to the phosphonium cation (P^+^) appear in the 2.0–4.0 ppm range. The peaks in this region are attributed to methylene protons directly connected to the phosphorus center, with the strong positive charge and heavy atom effect of phosphorus causing strong deshielding. By observing alkyl chain signals in the 0.8–1.5 ppm region, different chain lengths of phosphonium salts (hexadecyl > octyl > butyl) can be distinguished. Similar to ammonium catalysts, phosphonium catalysts also show significant residual signals, with TBHDPB (C16) showing the strongest alkyl chain signals, indicating substantial catalyst retention.

The XNBR samples obtained from different molecular weight PEG catalysts all show a sharp single peak at 3.6 ppm (Figure 2f), which is the characteristic signal of methylene protons in the PEG repeat unit (-O-CH_2_-CH_2_-O-). The peak shape of PEG-300-catalyzed samples shows certain broadening and irregularity; as molecular weight increases, the peaks become progressively sharper, with PEG-3000-catalyzed samples exhibiting extremely smooth and sharp single peaks. This phenomenon is attributed to the end-group effect. The proportion of hydroxyl protons at chain ends is relatively high in low molecular weight PEG, and these are prone to coupling with main chain protons, forming hydrogen bonds or being affected by trace moisture in the sample, resulting in spectral complexity. As molecular weight increases, the end group proportion drops sharply, and its influence becomes negligible. Importantly, all PEG-catalyzed samples show distinct PEG signals at 3.6 ppm, confirming that PEG cannot be completely removed from the XNBR product, which may affect material properties.

Comparing all catalysts, TBAB-catalyzed XNBR shows the cleanest ^1^H-NMR spectrum: the characteristic signals at 3.0–3.5 ppm are present but not excessively strong, indicating successful carboxylation without excessive catalyst retention. The alkyl chain signals in the 0.8–1.5 ppm region are moderate, consistent with its C4 chain length, and no additional peaks from catalyst residues are observed. This suggests that TBAB can be more effectively removed during product purification, yielding higher purity XNBR.

### 3.3. Gel Permeation Chromatography Analysis

**Figure 3:**
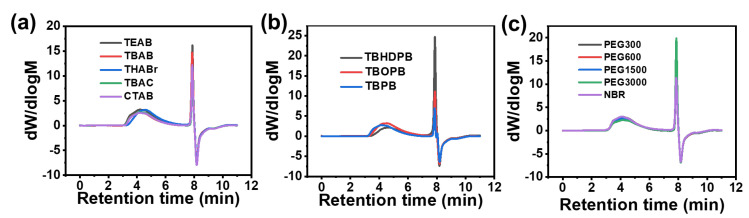
GPC curves of XNBR prepared with twelve different catalysts: (**a**) ammonium-based catalysts; (**b**) phosphonium-based catalysts; (**c**) PEG-based catalysts.

Gel Permeation Chromatography (GPC) (Figure 3, Table 1) provided insights into molecular chain size and distribution, revealing the significant influence of catalyst structure on the hydrolysis modification process. All carboxylated samples showed increased apparent molecular weights compared to unmodified NBR, but the extent of increase and the variation in molecular weight distribution (Đ, Mw/Mn) depended on catalyst type, clearly reflecting the efficiency and characteristics of different catalysts.

For quaternary ammonium and phosphonium catalysts, the results reveal the balance between catalytic activity and side reactions. Phosphonium catalysts (especially TBPB and TBHDPB) produced XNBR with the highest apparent Mw. This is consistent with their higher catalytic activity (due to the stronger polarizability of phosphorus atoms), where efficient hydrolysis introduces high-density carboxyl groups, generating the strongest hydrogen bond association effects [9]. However, GPC curves of these samples often show “shoulder peaks” in the high molecular weight region along with tailing or new peaks in the low molecular weight region, resulting in abnormally broadened molecular weight distribution (Đ). This strongly suggests that during hydrolysis, there may be slight polymer main chain degradation side reactions. Excessive basicity or nucleophilicity of catalysts, while attacking nitrile groups, may also cause damage to main-chain unsaturated bonds.

The influence of alkyl chain length: In both ammonium and phosphonium catalyst families, products prepared with long alkyl chain varieties (such as CTAB, THABr, TBHDPB) generally show higher apparent Mw than short-chain counterparts (such as TEAB). Long alkyl chains enhance catalyst compatibility and dispersion in the hydrophobic NBR matrix, improving catalytic efficiency. However, these samples also show broader molecular weight distributions and low molecular weight tailing, indicating that the reaction uniformity is compromised. The excessively long chains may lead to catalyst over-retention in the organic phase and localized over-reaction.

In the PEG series, XNBR prepared with PEG-600 and PEG-1500 exhibits the highest apparent Mw and the most significant molecular weight distribution broadening. This is attributed to their optimal catalytic efficiency, resulting in the highest degree of carboxylation. The introduction of numerous carboxyl groups forms strong intermolecular hydrogen bonds in the test solvent, triggering apparent association of macromolecular chains, thereby increasing measured hydrodynamic volume and Mw. The broadening reflects the heterogeneity of this association behavior among molecules of different chain lengths. In contrast, products from PEG-300 (insufficient catalytic activity) and PEG-3000 (reduced accessibility of catalytic sites due to steric hindrance) show smaller increases in apparent Mw, indicating limited carboxyl group introduction and weaker intermolecular association.

TBAB-catalyzed XNBR shows moderate apparent Mw increase (between short-chain TEAB and long-chain CTAB) and the narrowest molecular weight distribution. Its GPC curve is symmetrical with no abnormal shoulder peaks or low molecular weight tailing, confirming that TBAB achieves efficient carboxylation while effectively suppressing side reactions. The Đ value for TBAB-catalyzed XNBR is among the lowest of all catalysts, indicating the most uniform product structure. This superior molecular weight distribution control is a key factor in establishing TBAB as the optimal catalyst.

The molecular weight distribution data correlated well with the carboxyl content results. Phosphonium catalysts, particularly TBPB and TBHDPB, produced XNBR with the highest apparent Mw but also showed the broadest molecular weight distributions and significantly low molecular weight tailing, indicating that their high activity (carboxyl contents of 2.2 and 1.2 wt%) was accompanied by non-uniform reaction and possible degradation. Long-chain ammonium catalysts (CTAB, THABr) similarly showed broad distributions and low molecular weight tailing, consistent with their unexpectedly low carboxyl contents (0.7 and 0 wt%) due to catalyst over-retention and disrupted catalytic cycling. TBAB-catalyzed XNBR exhibited moderate Mw increase and the narrowest molecular weight distribution among all catalysts, with a symmetrical GPC curve and no abnormal shoulder peaks or low molecular weight tailing. This superior molecular weight distribution control, combined with its good carboxyl content (1.1 wt%) and clean product profile, further supports TBAB as the optimal catalyst.

### 3.4. Thermal Stability and Glass Transition Temperature Analysis

**Figure 4:**
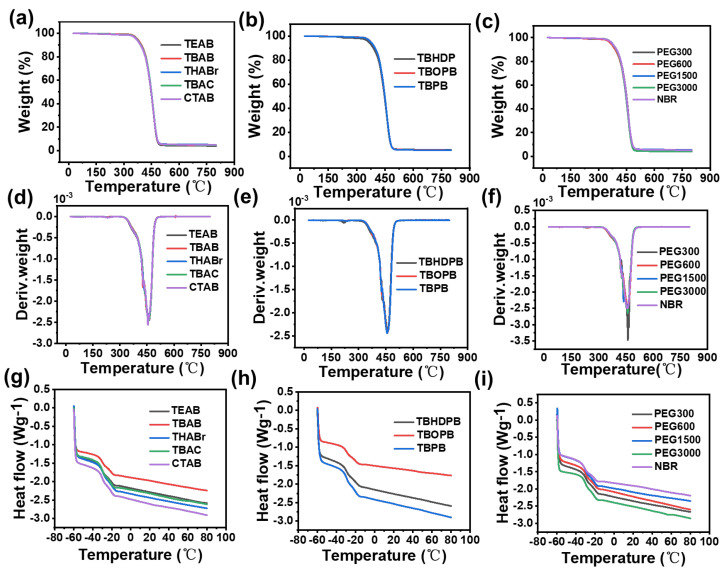
(**a**–**c**) TGA curves; (**d**–**f**) DTG curves; (**g**–**i**) DSC curves of XNBR prepared with twelve different catalysts.

Thermogravimetric analysis (TGA) was performed to evaluate the thermal stability of XNBR prepared using different catalyst systems. The TGA curves of all XNBR samples (Figure 4a–c) mainly presented two typical weight loss stages. The first stage, between approximately 300 and 400 °C, corresponded to the breakage and decomposition of the polymer main chain and represented the principal weight loss stage, while the second stage, between approximately 400 and 500 °C, corresponded to further oxidation and decomposition of residues. Compared with unmodified NBR, all carboxylated samples showed changes in decomposition onset temperature and residual carbon content, directly proving that carboxyl group introduction altered the thermal behavior of the polymer.

Among ammonium and phosphonium catalysts (Figure 4a,b), products from catalysts with long alkyl chains, such as THABr, CTAB, and TBHDPB, exhibited higher thermal stability and higher residual carbon content. Long alkyl chains not only enhanced catalytic efficiency by increasing carboxyl content but may also have participated in char formation at high temperatures, providing better insulation and barrier effects. However, this enhanced thermal stability came at the cost of broader molecular weight distributions and potential catalyst retention issues, as revealed by GPC analysis. When comparing catalysts with similar alkyl chain lengths, such as TBAB and TBPB, phosphonium-catalyzed XNBR typically showed slightly better thermal stability, which may be attributed to the higher catalytic efficiency of phosphonium catalysts, leading to more complete carboxylation and the potential flame-retardant effect of phosphorus, promoting the formation of more stable carbon layers at high temperatures. This observation correlates with the higher carboxyl contents achieved by phosphonium catalysts (TBPB: 2.2 wt%). Nevertheless, as noted earlier, phosphonium catalysts posed safety concerns due to violent reaction tendencies and showed significant catalyst retention, which may affect long-term material stability.

In the PEG series (Figure 4c), XNBR prepared with PEG-600 and PEG-1500 exhibited the best thermal stability, with onset decomposition temperature and maximum weight loss rate temperature significantly higher than those of other molecular weight PEG products. This was attributed to optimal molecular weight providing the best catalytic efficiency consistent with FTIR results and carboxyl content data (PEG-600: 0.6 wt%, PEG-1500: 0.2 wt%), resulting in moderate and uniform carboxylation that allowed the introduced carboxyl groups to form ionic cross-linking points in the early stage of thermal decomposition, thereby delaying main chain breakage. However, PEG-catalyzed samples showed additional low-temperature weight loss steps between 200 and 300 °C corresponding to residual PEG decomposition, confirming that PEG could not be completely removed and might compromise material purity despite the moderate carboxyl contents achieved. XNBR prepared with TBAB exhibited well-balanced thermal performance, with its onset decomposition temperature and maximum weight loss rate temperature at high levels comparable to or slightly better than most other catalysts, while its TGA curve was smooth and continuous with no additional low-temperature decomposition steps, indicating that TBAB-catalyzed hydrolysis achieved moderate and uniform carboxylation (1.1 wt%) with minimal catalyst residue and yielded XNBR with clean thermal decomposition behavior.

The corresponding DTG curves (Figure 4d–f) provided further insights into the decomposition kinetics. The maximum weight loss rate temperatures observed in the DTG profiles were consistent with the TGA data, with phosphonium-catalyzed samples showing slightly higher decomposition temperatures while PEG-catalyzed samples exhibited additional low-temperature peaks confirming residual PEG decomposition. Differential Scanning Calorimetry (DSC) analysis revealed that the glass transition temperatures (T_g_) of all XNBR samples prepared with different catalysts fell within a narrow range of approximately −28.5 °C to −25.9 °C (Figure 4g–i, Table S1). Compared with unmodified NBR (T_g_ = −28.5 °C), the introduction of carboxyl groups via phase transfer catalysis did not substantially alter the glass transition behavior, indicating that the catalyst systems and the resulting carboxylation had a limited impact on the overall chain segment mobility of the rubber. This suggests that the molecular flexibility essential for rubber elasticity was largely preserved across all catalyst systems evaluated.

### 3.5. Comprehensive Evaluation of Catalyst Systems

Based on the systematic characterization above, the twelve catalysts can be comprehensively evaluated across multiple dimensions. Long-chain ammonium catalysts (CTAB, THABr) show high initial catalytic efficiency due to excellent lipophilicity, as evidenced by their potential for strong carbonyl peaks in FTIR; however, their excessively long alkyl chains lead to catalyst over-retention in the organic phase, hindering continuous OH^−^ transport and creating steric hindrance effects, which is reflected in their low carboxyl contents (0–0.7 wt%), broader molecular weight distributions, and higher T_g_ values, indicating less uniform products and more rigid chain structures. TEAB, with its very short C2 chain, shows limited catalytic efficiency with only 0.2 wt% carboxyl content, while TBAB, with its moderate C4 chain, achieves the best balance with 1.1 wt% carboxyl content, narrowest molecular weight distribution, and lowest T_g_, making it clearly superior among ammonium catalysts.

Phosphonium catalysts show higher catalytic activity than their ammonium counterparts with similar chain lengths due to the stronger polarizability of phosphorus atoms, achieving the highest carboxyl contents (TBPB: 2.2 wt%, TBOPB: 1.4 wt%, TBHDPB: 1.2 wt%). TBPB with C4 chains shows high carboxylation efficiency but also the highest T_g_ among phosphonium catalysts, indicating more rigid chain structures. TBOPB with mixed butyl and octyl chains shows the lowest T_g_ due to internal plasticization effects, but its molecular weight distribution is broader, while TBHDPB with long C16 chains shows very high apparent Mw but extremely broad molecular weight distribution and significantly low molecular weight tailing, indicating non-uniform reaction and possible degradation. More importantly, phosphonium catalysts pose safety concerns due to their violent reaction tendencies with risks of localized overheating and runaway reactions, making them less suitable for practical applications despite their high activity.

PEG-300 and PEG-600 show moderate catalytic efficiency with carboxyl contents of 0.8 and 0.6 wt%, respectively, along with strong carbonyl peaks and reasonable thermal stability of the resulting XNBR. PEG-1500 and PEG-3000 show diminished efficiency with 0.2 and 0 wt% carboxyl content. However, a critical limitation of all PEG catalysts is the difficulty of complete PEG removal from the product, as all PEG-catalyzed samples show clear PEG signals in ^1^H-NMR and additional low-temperature decomposition steps in TGA, confirming residual PEG that may affect material purity, long-term stability, and performance especially in high-temperature applications.

Therefore, TBAB is established as the optimal catalyst system for phase transfer catalytic hydrolysis of NBR to prepare XNBR, as it uniquely combines strong catalytic efficiency (1.1 wt% carboxyl content, comparable to or better than most catalysts), mild and controllable reaction with no safety concerns, narrowest molecular weight distribution indicating uniform carboxylation with minimal side reactions, lowest T_g_ best preserving rubber elasticity, minimal catalyst retention with clean ^1^H-NMR spectrum, and simple, safe, reproducible reaction suitable for scale-up.

### 3.6. Performance Analysis of XNBR with Different Carboxyl Contents

Using TBAB as the catalyst and controlling reaction time (24 h and 48 h), two types of XNBR with different carboxyl contents were successfully prepared: low carboxyl content XNBR (IX-NBR, carboxyl content 1.1%) and high carboxyl content XNBR (HX-NBR, carboxyl content 3.1%). Their properties were systematically investigated.

**Figure 5:**
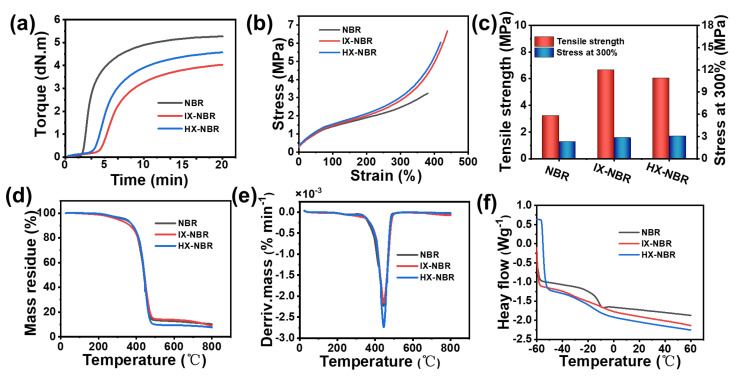
(**a**) Vulcanization curves; (**b**) stress-strain curves; (**c**) tensile strength and 300% modulus; (**d**) TG curves; (**e**) DTG curves; (**f**) DSC curves of NBR with different carboxyl contents.

Comparing the vulcanization curves and data of NBR, IX-NBR, and HX-NBR under the same formulation conditions (Figure 5a), the vulcanization time of IX-NBR was longer than that of NBR, increasing from 8.5 min for NBR to 13.0 min for IX-NBR (Table 2). As carboxyl content increased further, vulcanization time showed a trend of first prolonging and then shortening, decreasing to 11.4 min for HX-NBR. This is related to the inhibitory effect of carboxyl groups on the vulcanization process.

When carboxyl content in XNBR is low, carboxyl groups generate significant steric hindrance, preventing rubber molecular chains from approaching each other and making crosslinking point formation difficult, thus prolonging vulcanization time. When carboxyl content increases further, carboxyl groups can undergo acid-base reactions with zinc oxide (ZnO), consuming ZnO in the system. Additionally, carboxyl groups may even react with accelerators DM (dibenzothiazole disulfide) and TMTD (tetramethylthiuram disulfide), occupying original reaction sites and causing the vulcanization system to lose continuous reaction ability, thereby appropriately shortening vulcanization time [28,29].

The inhibitory effect of carboxyl groups on vulcanization was also reflected in maximum torque (MH). Due to consumption of the vulcanization system by carboxyl groups, the maximum MH after vulcanization decreased from 5.13 dN·m for NBR to 4.00 dN·m for IX-NBR. However, as carboxyl content further increased, new crosslink networks formed between carboxyl groups or between carboxyl groups and vulcanization additives, so MH of HX-NBR increased to 4.38 dN·m, though still lower than NBR.

The three NBR samples with different carboxyl contents were vulcanized, cut into dumbbell shapes, and subjected to mechanical property testing (Figure 5b). After carboxylation modification, the tensile strength of XNBR increased significantly. NBR exhibited a maximum tensile strength of only 3.1 ± 0.2 MPa and elongation at break of 391.1 ± 42.6% (Table 3). After carboxylation modification, tensile strength increased substantially. In IX-NBR, tensile strength and elongation at break increased to 6.6 ± 1.5 MPa and 438.3 ± 43%, respectively, indicating a more compact cross-linked network in the rubber system with increased crosslinking density.

XNBR essentially approaches a carboxylic acid-type ionic elastomer [30]. The leap in mechanical strength is mainly due to the enhanced effect of ionic clusters. When the material is subjected to external force, the highly reversible ionic crosslinking points (zinc carboxylate clusters) can effectively disperse and dissipate stress through a “dissociation-rearrangement-recombination” process, preventing rapid propagation of microcracks. At the same time, these nanoscale ionic clusters, as extremely powerful and uniformly dispersed physical crosslinking points, greatly enhance the modulus and strength of the material.

When carboxyl content increased further, both tensile strength and elongation at break of the rubber system decreased, with HX-NBR showing 6.0 ± 1.7 MPa and 418.8 ± 27.0%, respectively. This may be because at higher carboxyl content, the ionic crosslinking points in the system expand further, forming irreversible gel points that create stress concentration effects, ultimately leading to decreased mechanical properties.

Through TG and DTG analysis (Figure 5d,e), the mechanism of carboxylic acid content influence on thermal decomposition behavior of XNBR was systematically studied. As the carboxyl group content in NBR increases, the thermal stability of NBR gradually decreases (Table 4). First, in the initial stage of thermal decomposition, increased carboxyl content directly leads to a significant shift in T_5%_ (temperature at 5% weight loss). T_5%_ decreased from 343.3 °C for NBR to 340.5 °C for IX-NBR and 334.1 °C for HX-NBR. This phenomenon is attributed to the relatively weak thermal stability of carboxyl groups themselves. During programmed heating, carboxyl groups, as reactive sites, preferentially undergo dehydration or decarboxylation reactions at relatively lower temperatures (approximately 200–300 °C) compared to more stable nitrile groups (-CN) and the polymer main chain, escaping as small molecules. Therefore, higher carboxyl content leads to more significant pre-decomposition at low temperatures, macroscopically reflected in earlier deviation of the thermogravimetric curve from baseline and decreased onset decomposition temperature.

The 50% decomposition temperature (T_50%_) serves as an important indicator for measuring the thermal stability of composite rubber. After carboxylation modification, T_50%_ shows a slight increase, from 439.3 °C for NBR to 442.2 °C for IX-NBR and 441.3 °C for HX-NBR. This suggests that while carboxyl groups initiate decomposition earlier, the crosslinked network formed through ionic interactions provides some stabilization to the main chain during the core decomposition stage.

After carboxylation modification, the residual carbon content of NBR decreased from 10.1% in original NBR to 8.5% in IX-NBR and 7.3% in HX-NBR. For unmodified NBR during high-temperature pyrolysis, cyano groups tend to undergo crosslinking and cyclization reactions, forming a stable and dense gradient-like carbon-rich structure. Therefore, residual carbon content is relatively high. However, the introduced carboxyl groups have a profound negative impact on this process. On one hand, premature decomposition and gas escape of carboxyl groups leave defects on molecular chains, physically disrupting the continuity and density of potential stable carbon layers. On the other hand, decomposition reactions of carboxyl groups compete with carbonization reactions of cyano groups, chemically disrupting the formation of ordered carbon networks. Higher carboxyl content leads to stronger “damage” and “competition” effects, ultimately resulting in poorer quality and lower stability of the formed carbon layer, which is more prone to consumption at high temperatures. This is manifested as a significant reduction in final residual carbon content.

Through DSC systematic thermal analysis of XNBR with different carboxylic acid contents (Figure 5f), clear conclusions can be drawn: The *T*_g_ of XNBR has a significant dependence on carboxylic acid content. As carboxyl group content in the polymer chain increases, *T*_g_ systematically and continuously moves towards higher temperatures, indicating that transformation from the glassy state to the high elastic state requires higher energy input.

The direct physical essence of this phenomenon is that the introduction of carboxyl groups greatly limits the movement ability of polymer molecular chain segments. The fundamental reason lies in the formation of a strong, high-density intermolecular hydrogen bond network between carboxyl groups. These hydrogen bonds act as efficient physical crosslinking points in the rubber matrix, constructing a dynamic and reversible secondary crosslinking network at the molecular scale. This network significantly enhances interaction forces between molecular chains, thereby increasing the rigidity of the entire macromolecular chain, leading to a higher energy barrier for chain movement initiation, macroscopically manifested as a significant increase in *T*_g_.

Moreover, in XNBR samples with high carboxylic acid content, a broad endothermic relaxation peak can be observed above *T*_g_. This characteristic peak is attributed to the dissociation of the aforementioned dense hydrogen bond network during temperature increase. This phenomenon not only directly confirms the existence of strong hydrogen bond effects but also reveals the thermally reversible characteristics of the physical crosslinking network in XNBR.

## 4. Conclusions

In summary, this study systematically investigated the preparation of carboxylated nitrile rubber (XNBR) via phase-transfer catalytic hydrolysis using twelve catalysts across three categories: ammonium-based, phosphonium-based, and polyethylene glycols of different molecular weights. Through comprehensive characterization, all catalyst systems successfully introduced carboxyl groups onto NBR molecular chains, though efficiency varied significantly with catalyst structure. Long alkyl chain catalysts exhibited high initial efficiency but suffered from over-retention and broader molecular weight distributions. Phosphonium catalysts showed higher activity but posed safety concerns due to violent reactions. PEG catalysts with optimal molecular weight demonstrated excellent efficiency but could not be completely removed, affecting material purity. Tetrabutylammonium bromide (TBAB) was established as the optimal catalyst, achieving the best balance between catalytic efficiency and reaction controllability due to its moderate alkyl chain length and good leaving ability. Using TBAB, XNBR with low (1.1%) and high (3.1%) carboxyl contents were successfully prepared. Carboxyl content decisively influenced material properties: increased carboxyl content led to higher crosslink density and enhanced tensile strength (from 3.3 MPa for NBR to 6.7 MPa and 6.1 MPa for IX-NBR and HX-NBR, respectively), while elongation at break decreased. Thermal analysis showed decreased T_5%_ but improved T_50%_, and T_g_ increased systematically from −25 °C to 3.6 °C. This study provides important theoretical guidance for regulating carboxylation through catalyst molecular design and establishes TBAB-catalyzed phase transfer hydrolysis as a green, efficient approach for XNBR preparation with industrial potential.

## Figures and Tables

**Table 1:** Effects of different catalyst systems on the carboxyl content and molecular weight distribution of XNBR.

	-COOH (wt)	>1000 kDa	500–1000 kDa	300–500 kDa	200–300 kDa	100–200 kDa	50–100 kDa	10–50 kDa	<10 kDa
TEAB	1.1%	2.3	7.6	8.2	8.9	21.5	28.2	22.1	0.4
TBAB	0.7%	1.9	7.5	8.7	9.2	21.7	27.5	22.7	0.4
THABr	0.5%	0.2	2.2	4.8	6.9	20.7	32.8	31.8	0.8
TBAC	0.2%	1.8	6.9	8.0	8.7	21.2	28.1	23.7	1.0
CTAB	0.0%	1.5	6.8	8.2	8.9	21.5	28.3	23.9	0.5
TBHDPB	1.2%	0.005	0.9	3.1	5.4	19.9	34.1	35.9	0.7
TBOPB	1.4%	0.2	2.5	5.3	7.4	21.1	31.2	31.2	1.1
TBPB	2.2%	1.3	6.6	8.5	9.3	21.9	27.9	23.7	0.7
PEG300	0.8%	2.8	8.9	9.4	9.7	22.0	26.3	19.8	0.3
PEG600	0.6%	2.8	8.7	9.2	9.5	21.6	26.4	20.6	0.3
PEG1500	0.2%	2.6	8.5	9.1	9.5	21.7	26.2	20.8	0.5
PEG3000	0.0%	2.5	8.1	8.5	8.8	20.5	26.4	23.6	0.9
NBR	0.0%	2.7	8.9	9.7	10.1	22.5	25.8	18.8	0.3

**Table 2:** The curing characteristic parameters of XNBR with different carboxyl content.

Samples	t10 (min)	t90 (min)	ML (dN·m)	MH (dN·m)	△M (MH − ML) (dN·m)
NBR	2.3	8.5	0.04	5.13	5.09
IX-NBR	4.5	13.0	0.04	4.00	3.96
HX-NBR	3.7	11.4	0.03	4.38	4.35

t90 refers to the optimal vulcanization time (min), t10 refers to the scorch time (min), ML refers to the minimum torque (dN·m), MH refers to the maximum torque (dN·m), and △M refers to the difference between the maximum torque and the minimum torque (dN·m).

**Table 3:** Mechanical property parameters of XNBR with different carboxyl content.

Samples	Tensile Strength (MPa)	Elongation at Break (%)	Stress at 100% (MPa)	Stress at 300% (MPa)
NBR	3.1 ± 0.2	391.1 ± 42.6	1.3 ± 0.1	2.2 ± 0.1
IX-NBR	6.6 ± 1.5	438.3 ± 43.0	1.3 ± 0.1	2.7 ± 0.1
HX-NBR	6.0 ± 1.7	418.8 ± 27.0	1.5 ± 0.1	3.0 ± 0.2

**Table 4:** T_05%_, T_50%_, and char residue values of XNBR with different carboxyl content.

Samples	T_05%_ (°C)	T_50%_ (°C)	Char Residue Values (%)
NBR	343.3	439.3	10.1
IX-NBR	340.5	442.2	8.5
HX-NBR	334.1	441.3	7.3

## Data Availability

The data presented in this study are available on request from the corresponding author. Samples of the compounds are available from the authors.

## Supplementary Materials

The following supporting information can be downloaded at: https://www.mdpi.com/article/10.3390/polym18070830/s1, Figure S1. FT-IR and 1H-NMR spectra of NBR; Table S1. Glass transition temperatures of XNBR prepared with different catalysts and unmodified NBR.
