# Supplementary material for: Preparation and Performance Study of Carboxylated Nitrile Rubber Based on Phase Transfer Catalysis: Screening of Optimal Catalyst System
Source: Polymers (Basel). 2026 Mar 28;18(7):830. doi: 10.3390/polym18070830 (PMC13074642; doi:10.3390/polym18070830)
Supplement: Supplementary file 1 [file polymers-18-00830-s001.zip › polymers-4220947-supplementary.pdf]

Supporting Information

Preparation and Performance Study of Carboxylated Nitrile Rubber Based on Phase Transfer Catalysis: Screening of Opti-mal Catalyst System

Hongbing Zheng and Dongmei Yue*

Beijing University of Chemical Technology, Beijing 100029, PR China; Key Laboratory of Beijing City on Preparation and Processing of Novel Polymer Materials, Beijing 100029, P.R. China; zhenghongbing@petrochina.com.cn

* Correspondence: yuedm@mail.buct.edu.cn

Methods

1. ***Performance testing and characterization***

Infrared Spectroscopy: FTIR measurements were conducted using a Nicolet iS5 Fourier Transform Infrared Spectrometer (PerkinElmer, Waltham, MA, USA). The resolution was 4 cm^{-1}, and each spectrum was obtained through 32 scans over the wavenumber range of 4000 to 500 cm^{-1}.

^{1}H-NMR Spectroscopy: 5.0 mg of sample was dissolved in 1.0 mL of deuterated chloroform, and ^{1}H-NMR spectra were recorded using a Bruker 500 MHz nuclear magnetic resonance spectrometer to characterize the products obtained from different catalyst systems.

Thermal Stability Analysis: Thermogravimetric analysis (TGA) was performed using an STA 6000 (PerkinElmer, Norwalk, CT, USA) in a nitrogen atmosphere over the temperature range of 30 to 800 °C at a heating rate of 10 °C/min.

Differential Scanning Calorimetry (DSC): DSC measurements were conducted in a nitrogen atmosphere with a gas flow of 50.0 mL/min. Samples were heated from -60 °C to 80 °C at a heating rate of 10 K/min to determine the glass transition temperature (Tg) of the composite materials prepared with different catalysts.

Gel Permeation Chromatography (GPC): Molecular weight analysis was performed using a gel permeation chromatograph (GPC, Technologies 1260). 0.4-0.7 mg of sample was dissolved in chromatographic-grade toluene at a concentration of approximately 0.2-1 mg/mL. Rotorless Vulcanization: Vulcanization characteristics were measured using an MDR-U6S rotorless vulcanization instrument (Beijing Ruida Yuchen Instrument Co., Ltd.) at a temperature of 160 °C.

Mechanical Properties Testing: Mechanical properties were tested in accordance with national standard GB/T528-2009 using standard dumbbell-shaped samples (referencing GB/T 2941). Sample thickness was measured before testing, the test speed was 500 mm/min, and results were taken as the median of five parallel tests.

2. *Determination of carboxyl content in XNBR*

(1) Sample Dissolution: Approximately 0.20 ± 0.02 g of XNBR sample, was placed into a 250 mL stoppered conical flask containing a magnetic stir bar. 30 mL of pyridine was added using a measuring cylinder. The flask was placed on a magnetic stirrer and the mixture was stirred gently for at least 5 hours until complete dissolution was achieved.

(2) Titration: To the dissolved solution, 6-7 drops of thymolphthalein indicator solution were added (Thymolphthalein Indicator Solution (10 g/L): Weigh accurately 1.0 g of thymolphthalein and dissolve it in 95% ethanol. Dilute the solution to a final volume of 100 mL with additional 95% ethanol.). The solution was then titrated with standardized 0.05 mol/L potassium hydroxide in ethanol (KOH-ethanol) solution until a stable blue color appeared. The solution was allowed to stand for over 10 minutes. If the color faded to green or yellow within this period, titration was continued dropwise until the blue color reappeared and persisted unchanged for a further 10 minutes. The volume of KOH-ethanol titrant consumed was recorded as V (mL). A blank titration (without XNBR sample) was performed concurrently following the same procedure, and the volume consumed was recorded as V_0 (mL).

(3) Calculation: The carboxyl group content (X, expressed as mass percentage, wt%) in the XNBR was calculated using the following equation:

$X = [(V - V_0) \times c \times 45.03 \times 10^{-3}] / m \times 100\%$

c = Concentration of the KOH-ethanol standard titrant (mol/L)

V = Volume of KOH-ethanol titrant consumed by the sample (mL)

V_0 = Volume of KOH-ethanol titrant consumed by the blank (mL)

45.03 = Molar mass of the carboxyl group (-COOH) (g/mol)

m = Mass of the XNBR sample (g)

10^{-3} = Conversion factor from mL to L (to match concentration units)

The measurement results are presented as the average of five measurements.

The titration is based on the acid–base neutralization reaction: –COOH + KOH → –COOK + H_2O. Thymolphthalein is used as the indicator because its color transition (colorless to blue) occurs sharply in the pH range of 9.3–10.5, which is well above the complete neutralization endpoint of carboxyl groups. Under the experimental conditions (KOH-ethanol solution with phenolphthalein as indicator), the color

change from colorless to pink indicates that all free carboxyl groups have been fully neutralized, allowing accurate quantification of the carboxyl content.

3. *Mixing and vulcanization of XNBR/Lignin compounds*

Basic formula (by mass/ph): XNBR 100.0, zinc oxide 5.0, sulfur 1.5, stearic acid 1.0, DM 1.0, TMTD 0.2, totaling 148.7 parts.

Mixing process: At room temperature, start the water cooling system of the double-roll mill (X(S)K-160, First Rubber and Plastic Machinery Co., Ltd., Wuxi). First, wrap the raw rubber on a roll to plasticize it and then turn it over for 1 min. Then, gradually add sulfur, zinc oxide, and stearic acid. Rotate left and right alternately, turning the rubber over and making it wrapped/triangulated several times until all powders are fully mixed. Finally, add DM/TMTD and continue stirring until evenly distributed. The mixing time for each rubber sample is fixed at 15 min. After all rubber samples are mixed, increase the roller gap and pass each sample through the mill 10 times before discharging.

Vulcanization conditions: Place the rubber mixture at room temperature for 24 h. Cut a 5 g rubber sample into a round shape, measure the vulcanization curve at 165 °C using a rotorless vulcanization instrument (MDR-U6), obtaining parameters such as torque, scorch time (T10), and optimum vulcanization time (T90). Use an automatic rapid hot press molding machine (KSH-R-100T, Dongguan Kesheng Industrial Co., Ltd.) to vulcanize at 160 °C and 20 MPa for the vulcanization time T90 (min).

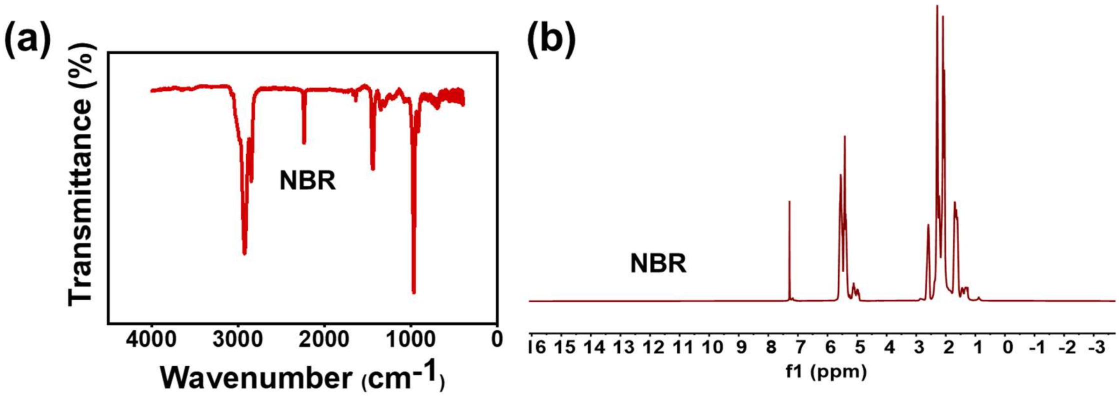

Figure S1. FT-IR and 1H-NMR spectra of NBR.

Table S1. Glass transition temperatures of XNBR prepared with different catalysts and unmodified NBR.

Catalyst	T_g (°C)
TEAB (Tetraethylammonium bromide)	-27.9
TBAB (Tetrabutylammonium bromide)	-27.7
THABr (Tetrahexylammonium bromide)	-26.7
TBAC (Tetrabutylammonium chloride)	-28.4
CTAB (Cetyltrimethylammonium bromide)	-27.8
TBHDPB (Tributylhexadecylphosphonium bromide)	-25.9
TBOPB (Tributyloctylphosphonium bromide)	-27.8
TBPB (Tetrabutylphosphonium bromide)	-27.2
PEG-300	-26.5
PEG-600	-26.8
PEG-1500	-27.8
PEG-3000	-28.4
Unmodified NBR	-28.5
